# Dietary Palygorskite Clay-Adsorbed Nano-ZnO Supplementation Improves the Intestinal Barrier Function of Weanling Pigs

**DOI:** 10.3389/fnut.2022.857898

**Published:** 2022-05-12

**Authors:** Lihuai Yu, Jun Liu, Junzhou Mao, Zhong Peng, Zhaoxing Zhong, Hongrong Wang, Li Dong

**Affiliations:** College of Animal Science and Technology, Yangzhou University, Yangzhou, China

**Keywords:** palygorskite clay-adsorbed nano-ZnO, immunity, barrier function, weaned pigs implications, intestinal morphology

## Abstract

This study aimed to investigate the effects of PNZ on intestinal mucosal barrier function in weaning piglets. A total of 210, 21-day-old piglets with similar body weights (6.30 ± 0.51 kg) were randomly allocated into seven groups: control group (CON), antibiotic group (ANT), ZnO group (ZO), nano-ZnO group (NZO) and low, middle, and high PNZ groups (LPNZ, MPNZ, and HPNZ). The seven groups were, respectively, fed control diets or control diets supplemented with antibiotics; 3,000 mg/kg ZnO; 800 mg/kg nano-ZnO; 700, 1,000, or 1,300 mg/kg PNZ. More integrated intestinal villi were observed in the LPNZ group. In the jejunum of LPNZ group, the crypt depth significantly decreased (*P* < 0.05), and the ratio of villus height to crypt depth (V/C) significantly increased (*P* < 0.05). In addition, the villus width and surface area of the ileum were significantly increased in the LPNZ group (*P* < 0.05). Dietary supplementation with PNZ can significantly increase the number of goblet cells in the mucosa of the jejunum and ileum (*P* < 0.05), decrease the contents of TNF-α and IL-1β (*P* < 0.05), and increase the contents of sIgA and IL-4 in the jejunal and ileal mucosa (*P* < 0.05). Meanwhile, the mRNA expression of MCU2 and ZO1 in PNZ group were significantly increased (*P* < 0.05), the mRNA expression of TLR4 and MyD88 was downregulated (*P* < 0.05). With increasing levels of PNZ, decreased proinflammatory cytokines and increased intestinal mucosal barrier function in weaned pigs was observed. In conclusion, supplementation with PNZ could effectively improve the intestinal barrier function of weanling piglets and potentially could replace the use of high doses of ZnO and antibiotics. The appropriate dose of PNZ for supplementation was 700 mg/kg.

## Implications

We identified a non-toxic, healthy feed additive that can effectively replace antibiotics and high zinc. Palygorskite clay-adsorbed nano-ZnO can effectively improve intestinal barrier function through improving intestinal morphology, increasing goblet cell numbers and immunoglobulin secretion and reducing pro-inflammatory cytokines secretion in weaning piglets. These findings provide a new feed additive for weaned piglets.

## Introduction

Antibiotics and high doses of ZnO are used in the livestock industry because of their ability to mitigate the adverse effects of weanling stress in piglets (1). However, researchers have found that the use of antibiotics has drawbacks, such as drug residues and bacterial resistance to antibiotics. Due to the low digestibility of zinc, high doses of ZnO in the feed leads to high zinc emission in feces of the livestock, thus causes the waste of resources and the environmental pollution (2, 3). Many countries have phased out the use of colistin sulfate, olaquindox and high-dose ZnO as feed additives. Therefore, seeking new, efficient, and green feed additives to replace the use of antibiotics and high doses of ZnO in the feed of livestock has become more and more important.

With the development of nanotechnology, nano-ZnO has been widely used in livestock science (4). Nano-ZnO has the characteristics of a large surface area and a high absorption rate (5) and it has a greater antibacterial effect than other feed additives (6). Nano-ZnO supplementation could improve the intestinal mucosal integrity in animals (7).

Palygorskite, also called attapulgite, is a kind of clay mineral with silicate as its main component. Palygorskite is non-toxic, odorless, non-irritating and has abundant reserves (8). The special structure of palygorskite gives it a high surface area, ion exchange and adsorption capacity as well as rheological and catalytic properties (9). Palygorskite can absorb heavy metal ions and mycotoxins and remove them from the animal (10, 11). In addition, a previous study suggested that clay minerals could be used as a controlled-release carrier for bioactive molecules, drugs and nutrients, thereby improving intestinal integrity, nutrient utilization and immune performance in livestock (12, 13). Hu et al. (14) reported that 600 and 900 mg/kg zinc oxide supported on a zeolite supplement improved growth performance and intestinal barrier function in weaned piglets. Hu et al. (15) demonstrated that supplementation with 500 and 750 mg/kg ZnO-montmorillonite alleviated post-weaning diarrhea and enhanced intestinal mucosal integrity.

Our previous studies showed that PNZ could improve the growth performance, decrease the diarrhea rate, and promote the growth and development of weaned piglets (16). We hypothesized that PNZ promoted weaned pig growth performance by improving the intestinal barrier function of piglets. In this study, we observed the samples by scanning electron microscopy, transmission electron microscopy and light microscopy and measured the length and width of the villi and the number of goblet cells. Then, the contents of immunoglobulin and immune cytokines in the jejunum and ileum were detected by ELISA kits, and the gene expression of pathway-related factors, immune cytokines and tight junction proteins in the jejunum and ileum was detected. Therefore, we investigated the possibility of PNZ substitution for antibiotics and high zinc and whether PNZ can improve the growth performance of weaned piglets by improving the intestinal barrier function.

## Materials and Methods

### Materials

The purity of ZnO is more than 98%. The purity of NZO is more than 99% and the average particle size of NZO is 45 nm. PNZ is comprised of 80% nano-zinc oxide and 20% palygorskite clay. The palygorskite clay is silicate with a double-layer chain structure, and the purity is 85%. The materials were all freely supplied by College of Chemical Engineering, Yangzhou University. The antibiotic consists of olaquindox (50%), chlortetracycline (15%), and colistin sulfate (10%), provided by Yangzhou University Feed Company. These three antibiotics are most widely used in piglet production to control intestinal infections (17, 18). The experimental piglets were 21-day-old Duroc-Landrace-Yorkshire weaned piglets provided by Suzhou Taicang Jinzhu Agricultural Development Co., Ltd.

#### Statement

This experiment were done in April 2017, shortly before the forbidden of colistin sulfate in the feed according to Announcement No. 2428 of the Ministry of Agriculture of the People’s Republic of China. The test time meets the standard of use. The antibiotics usage is limited to experimental research for the study of their substitutions.

### Animal Test

All procedures were approved by the Institutional Animal Care and Use Committee of Yangzhou University. A total of 210 21-day-old piglets (50% male and 50% female) with similar weights (6.30 ± 0.51 kg) were randomly allocated into seven groups, with six repetitions per group and five piglets per repetition. The groups were CON (control group, fed basal diets), ANT (antibiotic group, fed antibiotic-supplemented diets), ZO (ZnO group, fed 3,000 mg/kg ZnO-supplemented diets), NZO (nano-ZnO group, fed 800 mg/kg nano-ZnO-supplemented diets), LPNZ, MPNZ, and HPNZ (low, middle and high PNZ-supplemented groups, fed 700, 1,000, and 1,300 mg/kg PNZ-supplemented diets, respectively). The antibiotic group were fed by control diets supplemented with mixed antibiotics, including 100 mg/kg olaquindox (50%), 150 mg/kg chlortetracycline (15%), and 50 mg/kg colistin sulfate (10%).

The pig farm is a large-scale production facility, fully equipped, and takes complete biosecurity control and management measures. The experimental piglets were all raised in the same building. The temperature of the piggery is controlled at 25–30°C, the humidity is kept at about 65%. The pigs were gives 14 h illumination (6:00 a.m.–20:00 p.m.) everyday. The piglets had free access to food and water (fed four times a day, at 6:30, 10:30, 14:30, 18:30). The feeding experiment lasted 14 days. Diets were formulated according to the NRC (2012) (Table 1).

**TABLE 1:** Composition and nutrient levels of basal diets (air-dry basis).

Items	Content	Nutrition index^b^	Content
**Ingredient (%)**			
Corn	60.41	DE (MJ/kg)	13.39
Bran	4.00	CP %	20.45
Corn gluten meal	3.00	Ca %	0.85
Soybean oil	1.00	AP %	0.35
Soybean meal	28.00	Lys %	1.10
Limestone	1.26	Met+Cys %	0.69
CaHPO_4_	1.30	Thr %	0.78
*L*-Lys	0.15		
Met	0.02		
Phytase	0.01		
NaCl	0.35		
Premix^a^	0.50		
Total	100.00		

*^a^The premix provided the following per kilogram of the diet: VA 6,000 IU, VD_3_ 3,400 IU, VE 30 mg, VK_3_ 2 mg, VB_1_ 3.5 mg, VB_2_ 5.5 mg, VB_6_ 3.5 mg, VB_12_ 25.0 μg, biotin 0.05 mg, folic acid 0.3 mg, D-pantothenic acid 20 mg, niacin 20 mg, choline chloride 500 mg, Fe (as ferrous sulfate) 110 mg, Zn (as zinc sulfate) 100 mg, Cu (as copper sulfate) 20 mg, Mn (as manganese sulfate) 40 mg, Se (as sodium selenite) 0.30 mg, I (as potassium iodide) 0.40 mg.*

*^b^Nutrient levels were calculated values.*

### Sample Collection

On d 14 post-weaning (35 days), one piglet from each replicate was randomly selected, and the abdominal cavity was opened quickly after CO_2_ suffocation to separate the small intestine. The small intestine is separated from the pyloric sphincter distal 10 cm to the ileocecal fold proximal 10 cm. The final intersection point of the duodenum and pancreas was used as dividing point to separate the duodenum and jejunum. And the dividing point between the jejunum and ileum is 1/2 of the length of jejunum and ileum. The middle portion of jejunum and ileum at 1 cm was intercepted, which were fixed in 2.5% glutaraldehyde fixative solution for electron microscope observation. Then intestinal segments of 2 cm were cut from the middle part of jejunum and ileum, respectively. They were fixed in 4% paraformaldehyde for the study of intestinal morphology. The jejunum and ileum mucosa were gently scraped with slides and placed in a 2 ml cryo storage tube for preservation with liquid nitrogen. After the sampling, the samples were transferred to −80^°^C for testing.

### Histological Preparation

#### Electron Microscopy

The segments of intestine were used for scanning and transmission electron microscopy observation. Segments of the jejunum and ileum were taken from cryopreservation tubes, cut into approximately 1 mm^3^ tissue pieces, washed three times with 0.1 mol/L PBS, fixed in 1% citric acid for 2 h, washed with 0.1 mol/L PBS three times again, dehydrated with a gradient of ethanol and acetone, embedded, polymerized, and ultrathin sectioned. The treated tissue was observed under an electron microscope (Philips CM100, Royal Dutch Philips Electronics Ltd., Holland).

#### Optical Microscopy

Sample collection and processing were performed according to the operating method of Dong et al. (19). Tissue sections were observed and photographed by optical microscopy (Olympus IX53, Olympus Optical Co. Ltd., Tokyo, Japan). Two replicates were taken from each tissue of each replicate, the villus height (*v*), crypt depth and Villus width (*w*) of 10 different structures were measured under a microscope, and the villus surface area (*S*) was calculated using the following formula (19):


$$S=\pi \times \frac{w}{2}\times \sqrt{{\left(\frac{w}{2}\right)}^{2}+v^{2}}$$

#### Intestinal Epithelial Goblet Cells Study

Polysaccharide periodic acid-Schiff (PAS) staining was used to observe the number and distribution of goblet cells in the intestinal epithelium. Five samples were taken from each replicate for each tissue. The number of goblet cells per 100 intestinal mucosal epithelial cells of 10 intact villi was randomly observed and counted.

### Cytokine and Immunoglobulin Level Analysis by ELISA

The contents of IgA (sIgA), IgG, TNF-α, IL-1β, IL-4, and IL-10 in the jejunum and ileum were detected by ELISA kits. Samples of 250 mg of intestinal mucosa were mixed with 2.5 ml of total protein lysis buffer and homogenized in an ice box. Then, centrifugation was performed at 1,200 g/min for 10 min at 4^°^C. The supernatant was collected, and the content of total protein was determined by the BCA method in strict accordance with the kit’s operating procedures (Nanjing JianCheng Bioengineering Institute, Jiangsu, China). The immunoglobulin and cytokine concentrations in different samples were calculated by standard curves.

### mRNA Expression Measured by Real-Time PCR

Intestinal mucous membrane samples were taken from −80°C, the total RNA concentration of the sample was extracted using TRIzol (TIANGEN Biotechnology Co. Ltd., Beijing, China), the purity was determined (ABZ 7500), and the RNA quality was identified by agarose gel electrophoresis. The RNA was diluted to 500 μg/mL. A total of 800 ng of total RNA from each sample was reverse transcribed into cDNA in a 20 μL system. The specific protocol was performed according to the kit instructions (TAKARA RR047A).

Specific primers were designed and synthesized by GENEWIZ Biotech Co. Ltd. (Suzhou, China, Table 2). The relative expression levels of the target gene were compared with GAPDH, and the 2*^–ΔΔCT^* method was used to calculate the relative expression level of the target gene. RT-PCR was performed using the Applied Biosystems 7500 Fast Real-Time PCR System.

**TABLE 2:** Primer sequences used in quantitative real time PCR assays.

Gene	Accession no.	Primer sequence, sense/antisense	Amplicon size (bp)
GAPDH	NM_001206359	GTCGGAGTGAACGGATTTGGC GGAGGTCAATGAAGGGGTCA	106
TLR4	KF460453.1	GTGGCCTCCAAGGAACAAGA CTGGTGTTCACACGCACAAG	96
MyD88	EU056736.1	CTGGACATGGCAACTCCACA TTATAACCCAGCAAGCGGGC	116
NF-κB	EU399817.1	AGATCTTCCTGCTGTGCGAC GTCGGCTTGTGAAAAGGAGC	98
TNF-α	NM_214022	GCCCTTCCACCAACGTTTTC CAAGGGCTCTTGATGGCAGA	97
IL-1β	NM_001302388	GTGGCAGGACCTACACTCTTC TTCCTTCAGAATGCCGTCCTC	115
IL-4	NM_214123	CTCCCAACTGATCCCAACCC TGCACGAGTTCTTTCTCGCT	134
IL-10	NM_214041	GTGGCAGCCAGCATTAAGTC AACTCTTCACTGGGCCGAAG	103
MUC2	XM_021082584	CTGTGCGACTACAACTTCGC AGATGGTGTCGTCCTTGACC	139
ZO1	AJ318101	CTGAGGGAATTGGGCAGGAA TCACCAAAGGACTCAGCAGG	105
Occludin	NM_001163647	TGCCAGATCCTTCAACCCAC CCCTTGTGGCTCAGTGGAAA	92

### Statistical Analysis

Statistical analysis was performed by one-way analysis of variance (ANOVA) with SPSS software (Ver. 19.0 for windows, SPSS Inc., Chicago, IL, United States). Linear and quadratic contrasts were used to determine the effects of different doses of PNZ. Differences among groups were examined using Duncan’s multiple range tests. The results are expressed as means and pooled SEM. Statistical significance was set at *P* < 0.05, and 0.05 < *P* < 0.1 was discussed as trends.

## Results

### Effects of PNZ on the Intestinal Morphology of Weaned Pigs

**FIGURE 1:**
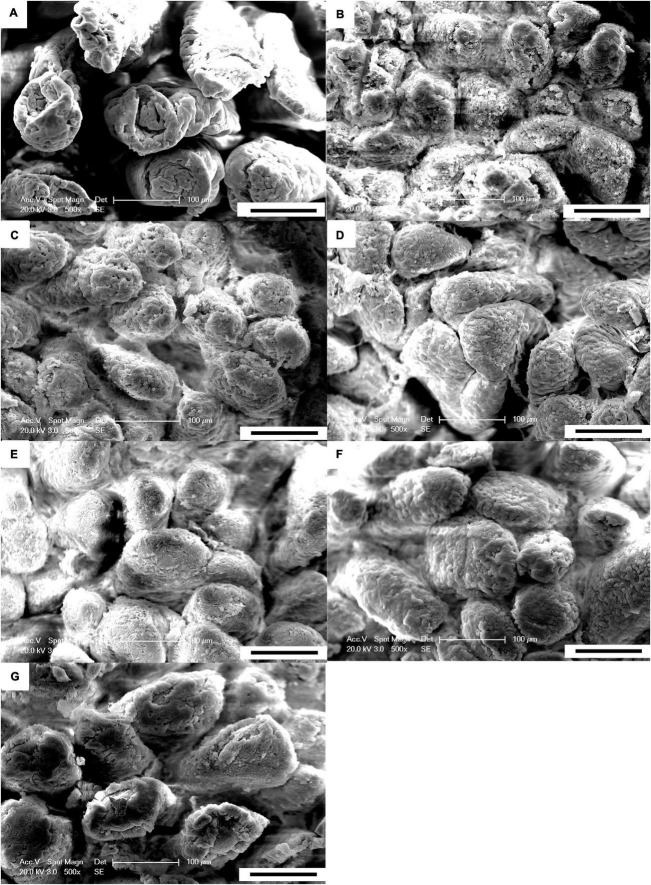
Scanning electron microscopy of the small intestine of weaned piglets (500×). **(A–G)** indicate the CON, ANT, ZO, NZO, LPNZ, MPNZ, and HPNZ groups, respectively. **(A–G)** show the villi magnified 500 times under a scanning electron microscope. The CON group has damaged, shorter and jagged intestinal villi in comparison with the PNZ group **(A,E–G)**. The integrity of the villi from the PNZ is greater than that of the ANT and NZO **(B,D)**. The villi structure of ZO is similar to that of PNZ **(C)**.

**FIGURE 2:**
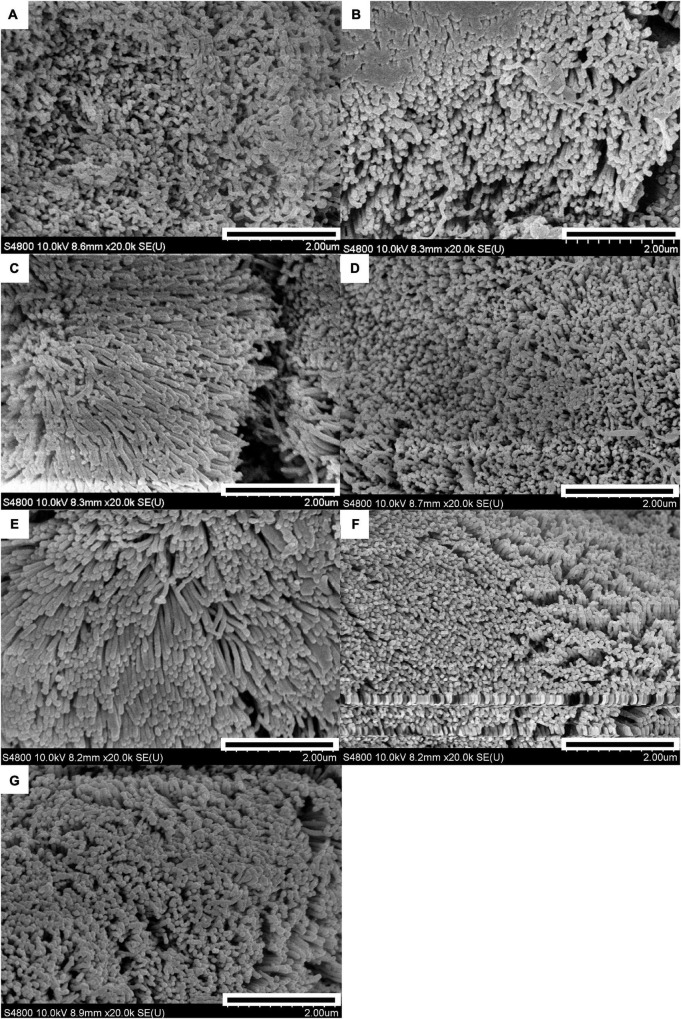
Scanning electron microscopy of the small intestine of weaned piglets (20 k). **(A–G)** indicate the CON, ANT, ZO, NZO, LPNZ, MPNZ, and HPNZ groups, respectively. **(A–G)** show the villi magnified 20 k times under a scanning electron microscope. Each group of villi is densely covered with microvilli. The microvilli of CON are sparse compared to the other groups, and there is no significant difference in microvilli development among the other groups **(A–G)**.

**FIGURE 3:**
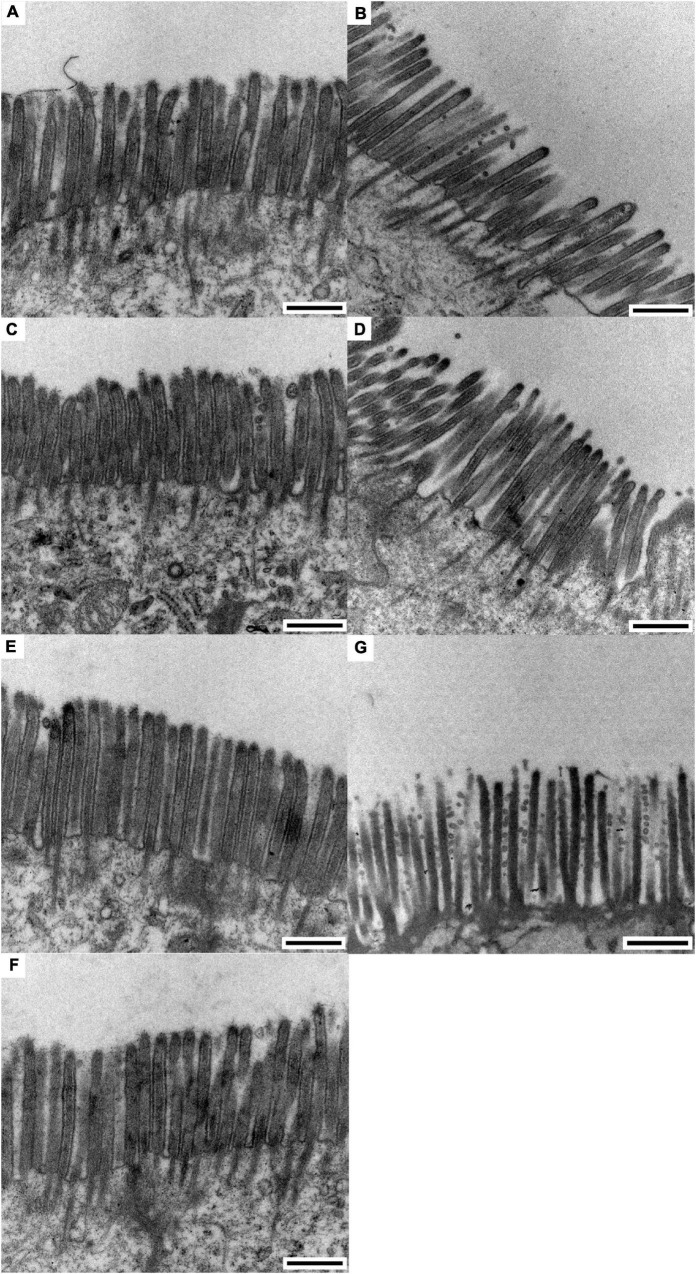
Transmission electron microscopy of the small intestine of weaned piglets (0.5 μm). **(A–G)** indicate the CON, ANT, ZO, NZO, LPNZ, MPNZ, and HPNZ groups, respectively. **(A–G)** show the appearance of the microvilli under a transmission electron microscope. Unit length 0.5 μm. The microvilli in the small intestine of CON, ANT, ZO, NZO, and HPNZ are damaged and differ in length **(A–D,G)**. In contrast, the microvilli of the LPNZ and MPNZ are arranged neatly, with uniform thickness and density **(E,F)**.

The results of the scanning electron microscopy observations are shown in Figures 1, 2. The CON (Figure 1A) showed damaged, shorter and jagged intestinal villi in comparison with PNZ (Figures 1E–G). The integrity of the villi from the PNZ was better than that of the ANT (Figure 1B) and NZO (Figure 1D). The villi structure of ZO (Figure 1C) was similar to the developmental level of PNZ. In addition, the microvilli of CON (Figure 2A) were sparse compared to the other groups, and there was no significant difference in microvilli development among the other groups (Figures 2B–G). The observations of the microvilli structure under microscopy are presented in Figure 3. The microvilli in the small intestine of CON, ANT, ZO, NZO, and HPNZ were damaged (Figures 3A–D,G). In contrast, the microvilli of LPNZ and MPNZ were undamaged and had a uniform density (Figures 3E,F).

As indicated in Table 3, the crypt depth in the jejunum of LPNZ in the weaned pigs was significantly lower than that of CON, ANT, and NZO (*P* < 0.05). Similarly, the ratio of villus height to crypt depth in the jejunum of LPNZ was significantly higher than that of CON and NZO (*P* < 0.05). Compared with the CON, ANT and NZO, the villus width of the ileum in ZO and LPNZ was significantly increased (*P* < 0.05); the villus surface area in the ileum of LPNZ and ZO were significantly higher than that of ANT and NZO (*P* < 0.05). The data showed that incremental levels of PNZ decreased the ratio of villus height to crypt depth in the jejunum (linear *P* = 0.010) and increased the villus width (linear *P* = 0.025) and villus surface (linear *P* = 0.018).

**TABLE 3:** Effects of PNZ on the intestinal morphology of the small intestine in weaned piglets.

Sites	Items	CON	ANT	ZO	NZO	PNZ^1^			SEM^2^	*P*-value	*P* ^3^	
						LPNZ	MPNZ	HPNZ			Linear	Quadratic
Jejunum	Villus length (μm)	442.42	526.51	536.10	487.63	561.71	514.45	476.67	11.50	0.09	0.029	0.879
	Crypt depth (μm)	153.78^a^	122.41^b^	119.82^bc^	126.33^b^	108.33^c^	123.71^b^	124.84^b^	2.56	<0.001	0.016	0.198
	Villi/crypt	2.99^c^	4.43^ab^	4.58^ab^	4.00^b^	5.32^a^	4.28^b^	3.88^bc^	0.16	0.001	0.010	0.459
	Villus width (μm)	66.88	69.00	72.56	66.20	72.79	69.24	66.29	1.06	0.440	0.104	0.927
	Villus surface area (mm^2^)	0.047	0.058	0.063	0.053	0.066	0.057	0.050	0.00	0.146	0.036	0.873
Ileum	Villus length (μm)	520.29	497.93	524.53	484.56	519.97	450.36	512.31	7.54	0.084	0.100	0.002
	Crypt depth (μm)	130.44	121.44	120.93	123.88	111.04	121.59	124.38	1.84	0.194	0.046	0.533
	Villi/crypt	4.11	4.18	4.42	4.06	4.81	3.76	4.23	0.10	0.119	0.032	0.913
	Villus width (μm)	65.54^b^	64.55^b^	74.04^a^	64.08^b^	72.86^a^	70.07^ab^	65.28^b^	1.02	0.013	0.025	0.709
	Villus surface area (mm^2^)	0.054^ab^	0.051^b^	0.061^a^	0.049^b^	0.061^a^	0.050^b^	0.052^ab^	0.00	0.021	0.018	0.430

*Data in the same column with different small letter superscripts mean significant difference (P < 0.05), while with the same or no letter superscripts mean no significant difference (P > 0.05). The same as below.*

*^1^PNZ, palygorskite clay adsorbed supplementation nano-ZnO, contain 80% Zn.*

*^2^Standard error of average value (n = 6).*

*^3^Effect of PNZ addition by polynomial contrasts.*

### Effects of PNZ on the Number of Goblet Cells in the Intestinal Mucosa of Weaned Pigs

Compared with CON and NZO, the number of goblet cells in the jejunum of ZO and LPNZ was significantly increased (*P* < 0.05), and the number of goblet cells in LPNZ was significantly higher than that in ANT (*P* < 0.05) (Table 4). In the MPNZ and ZO groups, the number of goblet cells in the ileum was increased compared to that in the CON group (*P* < 0.05), and the number of goblet cells in the ileum was greater in the MPNZ group than in the ANT group (*P* < 0.05). The number of goblet cells in the jejunum showed a linear (*P* < 0.001) increase with increasing PNZ inclusion in the diet.

**TABLE 4:** Effects of PNZ on the number of goblet cells in the intestinal mucosa of weaning piglets (per 100 columnar cells).

Sites	CON	ANT	ZO	NZO	PNZ^1^			SEM^2^	*P*-value	*P* ^3^	
					LPNZ	MPNZ	HPNZ			Linear	Quadratic
Jejunum	5.11^c^	5.60^bc^	6.25^ab^	5.37^c^	6.86^a^	5.78^bc^	5.10^c^	0.12	<0.001	<0.001	0.499
Ileum	5.86^c^	6.51^bc^	7.08^ab^	6.88^ab^	7.03^ab^	7.82^a^	6.99^ab^	0.14	0.007	0.176	0.477

*“/100 columnar cells” indicates the number of goblet cells per 100 epithelial columnar cells.^1^PNZ, palygorskite clay adsorbed supplementation nano-ZnO, contain 80% Zn.^2^Standard error of average value (n = 6).^3^Effect of PNZ addition by polynomial contrasts.*

### Effects of PNZ on the Contents of Cytokines and Immunoglobulins in the Intestines of Weaned Pigs

The contents of cytokines and immunoglobulins in the intestines of weaned pigs are presented in Tables 5, 6. For the jejunum, the concentration of TNF-α in the LPNZ was significantly lower than that in the CON, ANT, and NZO groups (*P* < 0.05). In addition, the content of IL-1β in the jejunum of PNZ was significantly lower than that of ANT and NZO (*P* < 0.05). Compared with the other groups, the contents of sIgA in the jejunum of the LPNZ were significantly increased (*P* < 0.05). The concentration of TNF-α in the jejunum increased linearly (*P* = 0.004), and the concentration of sIgA in the jejunum decreased linearly (*P* = 0.011) and quadratically (*P* = 0.046) with increasing PNZ levels. Compared with ANT, ZO, and NZO, the content of IL-4 in the ileum of LPNZ was significantly increased (*P* < 0.05). The content of IgG in the ileum of weaned pigs fed diets supplemented with ZO and NZO was significantly increased compared to that of CON, MPNZ, and HPNZ (*P* < 0.05). The content of IL-4 decreased linearly (*P* = 0.044) with increasing PNZ levels.

**TABLE 5:** Effects of PNZ on the contents of cytokines and immunoglobulins in jejunal mucosa of weaned pigs (ng/gprot).

Items	CON	ANT	ZO	NZO	PNZ^1^			SEM^2^	*P*-value	*P* ^3^	
					LPNZ	MPNZ	HPNZ			Linear	Quadratic
TNF-α	2.42^ab^	2.33^ab^	1.98^bc^	2.49^a^	1.79^c^	2.29^ab^	2.51^a^	0.07	0.014	0.004	0.435
IL-1β	5.13*^abc^*	6.44^a^	4.90*^bcd^*	6.22^ab^	3.82^cd^	4.70^cd^	3.51^d^	0.22	<0.001	0.575	0.044
IL-4	20.22	22.94	24.27	23.69	22.97	22.14	22.28	0.81	0.913	0.852	0.881
IL-10	3.33	3.46	3.69	3.49	3.80	3.78	3.41	0.05	0.120	0.024	0.213
sIgA (mg/mL)	10.33^b^	12.13^b^	11.65^b^	10.31^b^	13.93^a^	10.73^b^	11.15^b^	0.14	0.002	0.011	0.046
IgG (mg/mL)	76.76	77.81	73.28	79.70	78.86	81.15	87.62	1.23	0.081	0.023	0.503

*^1^PNZ, palygorskite clay adsorbed supplementation nano-ZnO, contain 80% Zn.^2^Standard error of average value (n = 6).^3^Effect of PNZ addition by polynomial contrasts.*

**TABLE 6:** Effects of PNZ on the contents of cytokines and immunoglobulins in ileal mucosa of weaned pigs (ng/gprot).

Items	CON	ANT	ZO	NZO	PNZ^1^			SEM^2^	*P*-value	*P* ^3^	
					LPNZ	MPNZ	HPNZ			Linear	Quadratic
TNF-α	1.53	1.40	1.42	1.49	1.44	1.33	1.54	0.06	0.977	0.875	0.466
IL-1β	5.17	5.33	4.83	5.57	4.67	5.02	5.20	0.17	0.870	0.547	0.909
IL-4	21.27^ab^	19.34^b^	20.27^b^	19.67^b^	24.20^a^	21.86^ab^	21.38^ab^	0.41	0.021	0.044	0.414
IL-10	2.21	2.53	2.47	2.73	2.79	2.68	2.46	0.06	0.167	0.203	0.758
sIgA (mg/mL)	5.52	6.07	5.76	5.93	6.81	6.25	6.22	0.19	0.673	0.403	0.666
IgG (mg/mL)	34.74^b^	38.64^ab^	42.26^a^	41.73^a^	36.48^ab^	32.88^b^	32.01^b^	0.96	0.011	0.072	0.458

*^1^PNZ, palygorskite clay adsorbed supplementation nano-ZnO, contain 80% Zn.^2^Standard error of average value (n = 6).^3^Effect of PNZ addition by polynomial contrasts.*

### Relative Gene Expression of Cytokines and Tight Junction Proteins in the Intestinal Mucosa of the Jejunum

As shown in Table 7, the mRNA expression of MUC2 in the jejunal mucosa of weaned pigs fed diets supplemented with LPNZ was significantly increased compared to that of CON, ANT, and NZO pigs (*P* < 0.05). In addition, compared with the other groups, the gene expression of ZO1 in the jejunal mucosa of LPNZ was significantly increased (*P* < 0.05). In contrast, the inclusion of ZO and MPNZ downregulated the expression of TNF-α in the jejunal mucosa (*P* < 0.05). The mRNA expression of MUC2 and ZO1 in the jejunal mucosa decreased linearly (*P* = 0.021, *P* = 0.022) with increasing PNZ.

**TABLE 7:** Effects of PNZ on the mRNA expression of jejunal mucosa of weaned pigs.

Items	CON	ANT	ZO	NZO	PNZ^1^			SEM^2^	*P*-value	*P* ^3^	
					LPNZ	MPNZ	HPNZ			Linear	Quadratic
TLR4	1.00	0.93	0.94	0.95	0.92	1.10	1.04	0.04	0.847	0.463	0.417
MyD88	1.00	0.86	1.05	0.99	0.98	0.93	0.93	0.03	0.763	0.698	0.854
NF-κB	1.00	0.89	0.95	0.87	0.99	0.91	0.89	0.04	0.960	0.582	0.859
TNF-α	1.00^ab^	0.81^bc^	0.68^c^	0.82*^abc^*	0.83*^abc^*	0.70^c^	1.06^a^	0.03	0.010	0.049	0.021
IL-1β	1.00	1.07	1.09	0.89	1.04	0.99	0.95	0.04	0.834	0.522	0.986
IL-4	1.00	0.98	0.97	0.91	0.99	0.88	0.95	0.03	0.936	0.718	0.274
IL-10	1.00	1.26	1.20	0.99	1.27	1.31	0.94	0.04	0.085	0.073	0.195
MUC2	1.00^b^	0.95^b^	1.34^ab^	0.91^b^	1.59^a^	1.10^b^	0.97^b^	0.06	0.034	0.021	0.389
Occludin	1.00	1.01	1.05	0.87	0.99	0.93	0.90	0.03	0.753	0.433	0.908
ZO1	1.00^b^	0.99^b^	1.03^b^	0.88^b^	1.45^a^	1.02^b^	1.00^b^	0.05	0.037	0.022	0.208

*^1^PNZ, palygorskite clay adsorbed supplementation nano-ZnO, contain 80% Zn.^2^Standard error of average value (n = 6).^3^Effect of PNZ addition by polynomial contrasts.*

### Relative Gene Expression of Cytokines and Tight Junction Proteins in the Intestinal Mucosa of the Ileum

As indicated in Table 8, in the ileum, the mRNA expression of TLR4 and MyD88 was downregulated in MPNZ compared with NZO (*P* < 0.05). Compared with ZO, the mRNA expression of TNF-α in the ileal mucosa of LPNZ and MPNZ was significantly decreased (*P* < 0.05). The mRNA expression of IL-10 and MUC2 in the ileal mucosa of weaned pigs fed diets supplemented with LPNZ was significantly increased compared to that of ANT and NZO (*P* < 0.05). As the PNZ level increased, the mRNA expression of TLR4 in the ileal mucosa increased linearly (*P* = 0.040 and *P* = 0.001) and quadratically (*P* = 0.011), and that of MUC2 decreased linearly (*P* < 0.001).

**TABLE 8:** Effects of PNZ on the mRNA expression of ileal mucosa of weaned pigs.

Items	CON	ANT	ZO	NZO	PNZ^1^			SEM^2^	*P*-value	*P* ^3^	
					LPNZ	MPNZ	HPNZ			Linear	Quadratic
TLR4	1.00*^abc^*	1.02^ab^	0.90*^abc^*	1.12^a^	0.71^bc^	0.67^c^	0.96*^abc^*	0.04	0.040	0.040	0.104
MyD88	1.00^ab^	0.81^bc^	0.87*^abc^*	1.03^ab^	0.69^c^	0.65^c^	1.08^a^	0.04	0.001	0.001	0.011
NF-κB	1.00	1.07	0.96	0.91	0.85	0.86	0.94	0.05	0.910	0.709	0.867
TNF-α	1.00^bc^	0.93^bc^	1.09^a^	0.98^bc^	0.84^bc^	0.71^c^	0.98^bc^	0.03	0.006	0.080	0.006
IL-1β	1.00	0.78	0.94	0.92	0.92	1.05	0.97	0.03	0.163	0.510	0.140
IL-4	1.00	0.96	0.93	0.92	0.99	1.00	0.95	0.04	0.996	0.614	0.655
IL-10	1.00^bc^	0.91^c^	1.10*^abc^*	0.96^c^	1.24^ab^	1.31^a^	1.06^bc^	0.04	0.010	0.159	0.147
MUC2	1.00^ab^	0.90^b^	1.04^ab^	0.87^b^	1.17^a^	0.95^b^	0.89^b^	0.03	0.013	<0.001	0.139
Occludin	1.00	0.94	0.99	1.17	0.97	1.04	0.94	0.02	0.083	0.641	0.122
ZO1	1.00	0.96	1.04	0.92	0.94	1.11	0.93	0.02	0.345	0.876	0.005

*^1^PNZ, palygorskite clay adsorbed supplementation nano-ZnO, contain 80% Zn.^2^Standard error of average value (n = 6).^3^Effect of PNZ addition by polynomial contrasts.*

## Discussion

Weaning influences the intestinal mucosal immunity and growth performance of pigs (20). Intestinal mucosal immunity is an important factor for the intestinal development of pigs. Generally, to relieve weaning stress in production, the addition of zinc oxide and antibiotics has a good effect (21). However, zinc has a low digestibility in animals, and a high dose of zinc fed to animals will lead to a large amount of zinc excretion in feces, resulting in a waste of resources and environmental pollution (22). The European Union and the United States have already restricted the amount of zinc that can be added to piglet feeds. Therefore, it is urgent to reduce the amount of zinc in feed and the use of antibiotics.

In recent years, it has been reported that clay minerals could be used as controlled-release carriers for bioactive molecules, drugs and nutrients (23). Feeding clay minerals can protect the integrity of intestinal villi and improve intestinal immunity (8). Therefore, we studied the effect of palygorskite on the intestinal membrane immune function of weaned piglets with the goal of reducing zinc and replacing antibiotics.

In the current study, the results of electron microscopy observation showed that the integrity of the intestinal villi of PNZ was better developed. Zhang et al. confirmed that dietary supplementation with palygorskite improved the ileal villus height and villus diameter in weanling piglets (24). Long et al. showed that dietary supplementation with nano-ZnO decreased the crypt depth of the jejunum and ileum and improved the integrity of the intestine (25). The results of this experiment were similar to theirs. In addition, our present study found that the intestinal morphology was better in the 700 mg/kg PNZ group than in the 800 mg/kg nano-ZnO group. This result supports the conclusion that palygorskite could enhance the intestinal development and integrity of weaned pigs.

PNZ improved the residence time and utilization rate of feed ingredients in the intestine (26). The harmful toxins and fungal substances in the intestine and feed-stuffs could be adsorbed by palygorskite, and then be excreted to the outside of the body (27). However, previous studies have also shown that excessive amounts of palygorskite in feed could reduce digestive enzyme activity and nutrient digestibility in the intestine (28). Similarly, a previous study has shown that dietary supplementation of 5,000 mg/kg nano-zno can impair liver function and alter zinc metabolism in the small intestine (29). Therefore, high-dose PNZ may hinder the intestinal absorption of various nutrients, thereby inhibiting intestinal development. Our previous study indicated that 700 mg/kg PNZ effectively improved the growth performance of weaned piglets and reduced the diarrhea rate, which was in accordance with the results of this study (16).

Tight junction proteins and mucins affect intestinal mucosal integrity (30). ZO1 is a tight junction protein that connects cells to form a barrier to protect the body from the contents of the intestine (31). The current study showed that dietary supplementation with 700 mg/kg PNZ could upregulate the gene expression of ZO1 better than ANT and NZO, which indicated that PNZ has a beneficial effect on barrier function and intestinal integrity. Cheng et al. (13) also found that dietary supplementation with palygorskite upregulated the gene expression of ZO1 in the intestine of broilers and improved their intestinal integrity and barrier function. Goblet cells are specialized cells that can secrete multiple mucins (32). The mucus layer is formed by mucins combined with water, inorganic salts, and antimicrobial peptides, which could protect the gastrointestinal tract from invading pathogens and promote the development of the intestine (33). MUC2 is the main protein component of the intestinal mucus and can provide a physical barrier between the epithelium and the luminal content (34). The results of this study showed that the number of goblet cells in the intestine of the PNZ- and ZO-supplemented groups was increased. Similarly, the mRNA expression of MUC2 in the intestine of PNZ was increased. Previous studies have shown that both of nano-ZnO and palygorskite could increase the number of goblet cells in the intestine (24, 35). Palygorskite supplementation increased the number of goblet cells and the secretion of mucins, such as MUC2, in the intestine and improved the intestinal injury resistance and villus development of broilers (13). The results of our study were in accordance with these studies.

Goblet cells secrete mucin to isolate pathogenic bacteria (36). And with a high surface area, PNZ can be a sorbent for bacteria and even viruses (37). In this study, PNZ increased of the number of goblet cells, effectively protected the intestinal mucosal integrity and promoted villus development. The modulatory effects of PNZ on intestinal goblet cell number and mucin secretion may be related to both the effects of palygorskite and NZO on the intestinal mucosa (38). Thus, PNZ may affect the mRNA expression of tight junction proteins due to the effect of clay minerals, and the increased mRNA expression of mucin may be due to an improvement in the number of goblet cells. The mechanism by which PNZ regulates the mRNA expression of mucin and tight junction proteins in weaned pigs needs further study.

The function and permeability of the small intestine is influenced by immunoglobulins and a variety of cytokines, including interferon and interleukins (39). These pro-inflammatory cytokines may affect intestinal mucosal epithelial cells and influence the integrity and function of the intestine (40). After weaning, the levels of inflammatory cytokines in the small intestine show different expression levels (41). Excessive proinflammatory cytokines cause inflammation of the intestines and impair the health of the intestine (42). TNF-α and IL-1β have been confirmed to be proinflammatory cytokines (43); IL-4 and IL-10 are anti-inflammatory factors that regulate immune responses through TLR4, NF-κB, and other signaling pathways to maintain intestinal health (44). IgA and IgG may activate phagocytosis of macrophages to remove antigens and prevent pathogens from damaging the mucosal barrier (45).

The results of this study showed that PNZ supplementation reduced the concentration of proinflammatory cytokines and increased the concentrations of sIgA and IgG in the intestine of weaned piglets, and we found that dietary supplementation with 3,000 mg/kg ZnO had a better inhibitory effect on IL-1β. Palygorskite can regulate the immune system and increase the phagocytic activities of polymorphonuclear leukocytes and serum antibody production in mice (46). The NZO and MPNZ groups contained the same concentration of nano-ZnO, but the MPNZ group had better effects on improving the secretion of cytokines and immunoglobulins. This might be attributed to the role of palygorskite. In addition, the interaction mechanism between palygorskite and zinc oxide is unknown.

Stimulation of the extracellular domain of TLR triggers the intracellular association of MyD88 with its cytosolic domain for the activation of NF-κB, which ultimately leads to the synthesis and release of a number of proinflammatory mediators (47). In this study, PNZ supplementation downregulated the gene expression of TLR4, MyD88, and TNF-α. We inferred that TLR4 regulated the expression of MyD88 and then decreased the content of NF-κB to inhibit the mRNA expression of proinflammatory cytokines. In addition, the mRNA expression of IL-10 was upregulated. The mRNA expression of TLR4 and MyD88 observed in the present study was in agreement with the results of Cheng et al., who found that dietary palygorskite supplementation improved intestinal immune function of weaned piglets by downregulating the expression of IL-1β and IFN-γ in the TLR4 pathway (13). Hu et al. showed that zinc oxide supported on zeolite decreased the mRNA levels of TNF-α and IFN-γ on Day 7 post-weanling of pigs (14). Furthermore, the present results of PNZ increasing the mRNA levels of IL-10 in weaned pigs were consistent with Chao et al. (48). Thus, the improved intestinal integrity and barrier function of weaning piglets might be associated with downregulated expression of the TLR4 signaling pathway.

## Conclusion

Compared with the ZnO group and the Ant group, the PNZ groups exhibited improved intestinal villus development, goblet cells and immunoglobulin secretion, decreased contents and mRNA expression of proinflammatory cytokines, and increased tight junction protein mRNA expression in weaned pigs. These results indicated that PNZ could be used as a substitute for ZnO and ANT in weaning pigs. The results showed that the best feeding effect was at 700 mg/kg.

## Data Availability Statement

The raw data supporting the conclusions of this article will be made available by the authors, without undue reservation.

## Ethics Statement

The animal study was reviewed and approved by the Ethical Review Committee of experimental animal welfare of Yangzhou University.

## Author Contributions

LY and LD conceived and designed the research. JL and JM conducted the experiments and performed data analysis. JL and LY wrote the manuscript. LD revised the manuscript. All authors read and approved the manuscript.

## Conflict of Interest

The authors declare that the research was conducted in the absence of any commercial or financial relationships that could be construed as a potential conflict of interest.

## Publisher’s Note

All claims expressed in this article are solely those of the authors and do not necessarily represent those of their affiliated organizations, or those of the publisher, the editors and the reviewers. Any product that may be evaluated in this article, or claim that may be made by its manufacturer, is not guaranteed or endorsed by the publisher.

## Abbreviations

PNZ: Palygorskite clay adsorbed nano-ZnO
CON: control group
ANT: antibiotic group
ZO: ZnO group
NZO: nano-ZnO group
LPNZ: low PNZ supplemented group
MPNZ: middle PNZ supplemented group
HPNZ: high PNZ supplemented group
V/C: the ratio of villus length vs. crypt depth
sIgA: secretory immunoglobulin A
IL: interleukin
TNF-α: tumor necrosis factor-α
MUC2: mucoprotein 2
ZO1: zonula occludens 1
TLR4: Toll Like Receptor 4
MyD88: myeloid differentiation factor 88
PBS: phosphate buffered saline
IgG: immunoglobulin G
SPSS: Statistical Package for Social Sciences.
